# Supplementary material for: Metabolic signatures for gastric cancer diagnosis and mechanistic insights: a multicenter study
Source: EMBO Mol Med. 2025 Oct 27;17(12):3355–76. doi: 10.1038/s44321-025-00325-0 (PMC12686445; doi:10.1038/s44321-025-00325-0)
Supplement: Supplementary file 1 — Appendix [file 44321_2025_325_MOESM1_ESM.pdf]

Appendix

Appendix Figure S1. Kyoto Encyclopedia of Genes and Genomes (KEGG) metabolic pathways enriched by 26 replicated differential metabolites between GC patients and non-GC controls in the discovery and validation dataset. 2

Appendix Figure S2. Permutation test for model overfitting ($p < 0.001$)...............................3

Appendix Figure S3. The boxplot demonstrated six metabolites were significantly altered in GC ascompared with non-GC in both the discovery and validation dataset.........................4

Appendix Table S1. 26 candidate blood metabolites in the discovery and validation dataset..5

Appendix Table S2. The FCs, FDR and AUC (95%CI) of 26 candidate blood metabolites in the discovery and validation dataset. 6

Appendix Table S3. LRScores of 26 selected features. 7

Appendix Table S4. The diagnostic performance of 26 replicated blood metabolites in the discovery and validation dataset. 8

Appendix Table S5. Average diagnostic scores of 26 metabolites in the discovery dataset and validation dataset. 9

Appendix Table S6. Average diagnostic scores of six metabolic biomarkers in the discovery and validation dataset. 10

Appendix Table S7. The diagnostic sensitivity of metabolic biomarker panel compared with clinical protein biomarker tests. 11

Appendix Table S8. The diagnostic performance of six metabolic biomarkers in the discovery and validation dataset, adjusting age, sex, smoking and drinking. 12

Appendix Table S9. Details of the genome-wide association studies and datasets used in MR analyses.. 13

Appendix Table S10. Characteristics of the genetic variants associated with the circulating level of isovalerylcarnitine (C5). 14

Appendix Table S11. N-acetylneuraminate showed no statistically significant association with GC in Mendelian randomization analyses. 15

Appendix Table S12. Genetic variants associated with the circulating level of isovalerylcarnitine (C5) in reverse Mendelian randomization analyses... 16

Appendix Table S13. Differentially expressed proteins significantly correlated with isovalerylcarnitine (C5) expression. 17

Appendix Table S14. Sensitivity analysis on AUC across different missing value imputation methods. 20

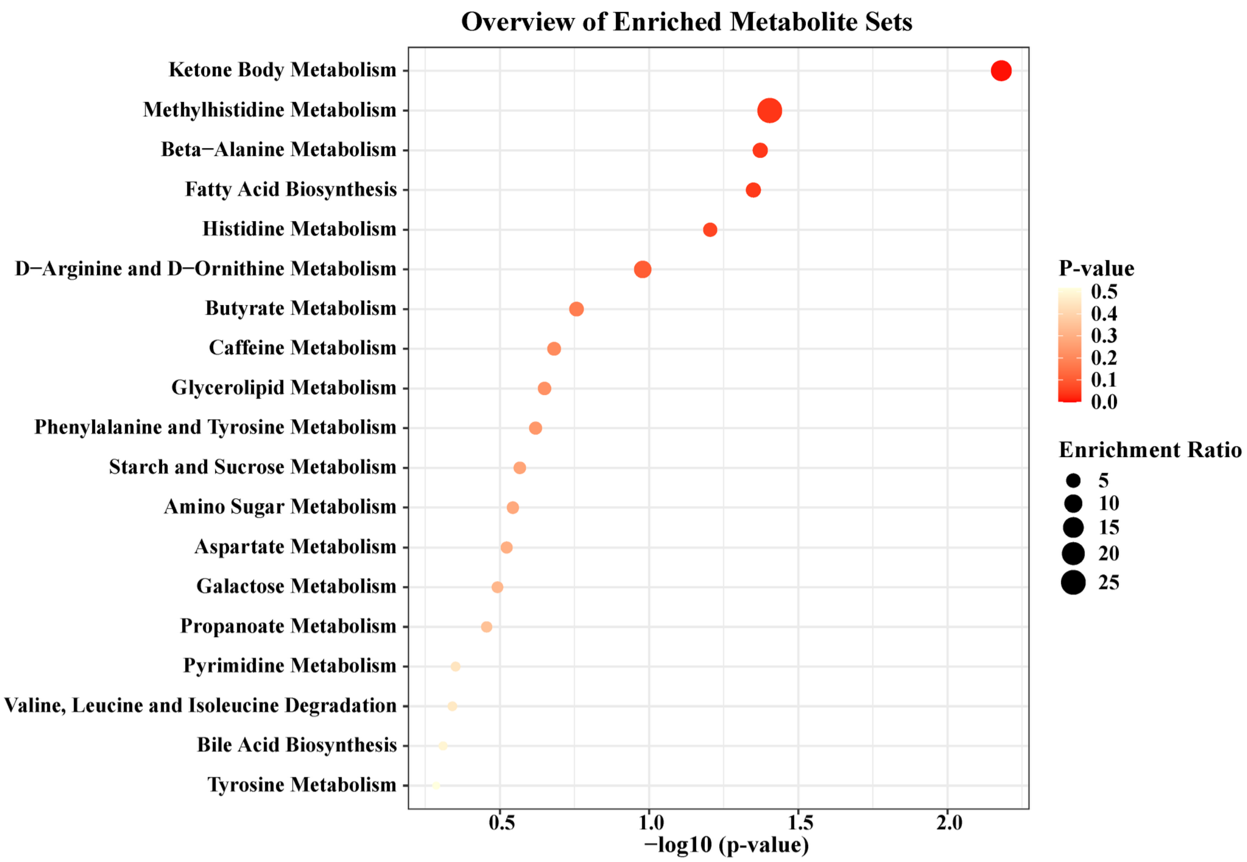

Appendix Figure S1. Kyoto Encyclopedia of Genes and Genomes (KEGG) metabolic pathways enriched by 26 replicated differential metabolites between GC patients and non-GC controls in the discovery and validation dataset.

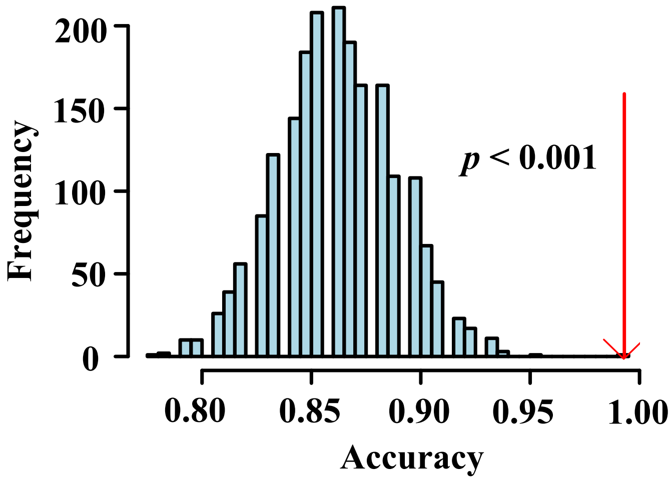

Appendix Figure S2. Permutation test for model overfitting ($p < 0.001$).

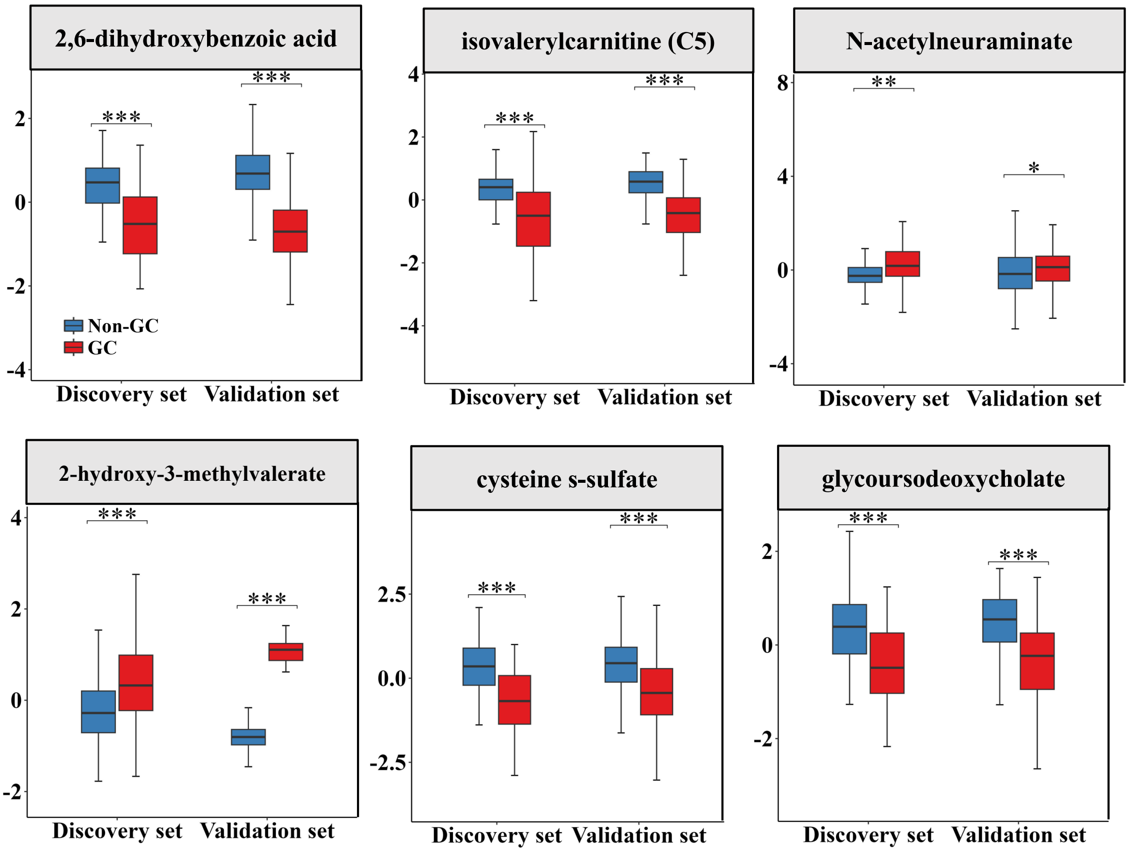

Appendix Figure S3. The boxplot demonstrated six metabolites were significantly altered in GC as compared with non-GC in both the discovery and validation dataset.

Appendix Table S1. 26 candidate blood metabolites in the discovery and validation dataset.

Index	COMPOUND Name	Molecular formula	HMDB ID	Super meta pathway	Sub meta pathway
1	N-acetylneuraminate	C11H19NO9	HMDB0000230	Carbohydrate	Aminosugar Metabolism
2	acisoga	C9H16N2O2	HMDB0061384	Amino Acid	Polyamine Metabolism
3	3-hydroxybutyrate (BHBA)	C4H8O3	HMDB0000011	Lipid	Ketone Bodies
4	2-hydroxy-3-methylvalerate	C6H12O3	HMDB0000317	Amino Acid	Leucine, Isoleucine and Valine Metabolism
5	isovalerylcarnitine (C5)	C12H23NO4	HMDB0000688	Amino Acid	Leucine, Isoleucine and Valine Metabolism
6	cysteine s-sulfate	C3H7NO5S2	HMDB0000731	Amino Acid	Methionine, Cysteine, SAM and Taurine Metabolism
7	glycoursodeoxycholate	C26H43NO5	HMDB0000708	Lipid	Secondary Bile Acid Metabolism
8	2,6-dihydroxybenzoic acid	C7H6O4	HMDB0013676	Xenobiotics	Drug - Topical Agents
9	pentose acid**	C5H10O2	HMDB0000892	Partially Characterized Molecules	Partially Characterized Molecules
10	oxindolylalanine	C11H12N2O3		Amino Acid	Tryptophan Metabolism
11	tiglylcarnitine (C5:1-DC)	C12H21NO4	HMDB0002366	Amino Acid	Leucine, Isoleucine and Valine Metabolism
12	linoleoyl-arachidonoyl-glycerol (18:2/20:4) [2]*	C41H68O5	HMDB0007257	Lipid	Diacylglycerol
13	oleoyl-arachidonoyl-glycerol (18:1/20:4) [2]*	C41H70O5	HMDB0007228	Lipid	Diacylglycerol
14	C-glycosyltryptophan	C17H22N2O7	HMDB0240296	Amino Acid	Tryptophan Metabolism
15	N6-succinyladenosine	C14H17N5O8	HMDB0000912	Nucleotide	Purine Metabolism, Adenine containing
16	glycerol	C3H8O3	HMDB0000131	Lipid	Glycerolipid Metabolism
17	acetoacetate	C4H6O3	HMDB0000060	Lipid	Ketone Bodies
18	hexanoylglutamine	C11H20N2O4	HMDB0242182	Lipid	Fatty Acid Metabolism (Acyl Glutamine)
19	maltose	C12H22O11	HMDB0000163	Carbohydrate	Glycogen Metabolism
20	glycochenodeoxycholate	C26H43NO5	HMDB0000637	Lipid	Primary Bile Acid Metabolism
21	1-methylurate	C6H6N4O3	HMDB0003099	Xenobiotics	Xanthine Metabolism
22	2-oxoarginine*	C6H11N3O3	HMDB0004225	Amino Acid	Urea cycle; Arginine and Proline Metabolism
23	androstenediol (3beta,17beta) monosulfate (1)	C19H30O5S	HMDB0240429	Lipid	Androgenic Steroids
24	2,3-dihydroxyisovalerate	C5H10O4	HMDB0012141	Xenobiotics	Food Component/Plant
25	beta-alanine	C3H7NO2	HMDB0000056	Nucleotide	Pyrimidine Metabolism, Uracil containing
26	3-methylhistidine	C7H11N3O2	HMDB0000479	Amino Acid	Histidine Metabolism

Appendix Table S2. The FCs, FDR and AUC (95%CI) of 26 candidate blood metabolites in the discovery and validation dataset.

Replicated metabolites		Discovery dataset			Validation dataset		
		FC	FDR	AUC(95%CI)	FC	FDR	AUC(95%CI)
1	maltose	8.77	6.76E-04	0.631(0.540-0.721)	12.75	1.27E-29	0.799(0.758-0.839)
2	3-hydroxybutyrate (BHBA)	3.14	7.56E-06	0.730(0.647-0.813)	3.64	5.73E-58	0.874(0.842-0.906)
3	hexanoylglutamine	2.68	8.67E-06	0.751(0.669-0.834)	3.24	1.20E-38	0.832(0.795-0.869)
4	acetoacetate	2.38	1.78E-02	0.695(0.608-0.782)	1.33	3.05E-09	0.663(0.613-0.712)
5	N6-succinyladenosine	1.80	3.35E-02	0.637(0.543-0.730)	1.05	5.53E-03	0.593(0.541-0.646)
6	glycerol	1.71	6.81E-07	0.746(0.667-0.825)	1.26	1.13E-07	0.638(0.588-0.689)
7	2-hydroxy-3-methylvalerate	1.66	6.76E-04	0.690(0.601-0.778)	1.20	1.20E-06	0.616(0.564-0.667)
8	C-glycosyltryptophan	1.65	5.35E-03	0.679(0.591-0.768)	1.13	1.06E-04	0.598(0.546-0.650)
9	N-acetylneuraminate	1.63	2.78E-03	0.661(0.572-0.751)	1.11	2.86E-03	0.595(0.543-0.648)
10	acisoga	1.57	5.59E-03	0.665(0.575-0.755)	1.20	9.43E-05	0.601(0.549-0.653)
11	glycoursodeoxycholate	0.33	1.73E-06	0.741(0.658-0.824)	0.37	1.66E-22	0.768(0.725-0.811)
12	2,6-dihydroxybenzoic acid	0.43	4.04E-10	0.815(0.745-0.885)	0.29	3.57E-65	0.895(0.865-0.924)
13	glycochenodeoxycholate	0.52	1.10E-02	0.632(0.540-0.724)	0.48	2.68E-17	0.728(0.682-0.774)
14	pentose acid**	0.52	1.89E-08	0.752(0.675-0.829)	0.90	5.95E-05	0.554(0.501-0.607)
15	cysteine s-sulfate	0.52	1.81E-09	0.796(0.724-0.868)	0.71	7.47E-17	0.702(0.653-0.750)
16	oxindolylalanine	0.56	3.28E-14	0.855(0.793-0.918)	0.87	1.42E-03	0.568(0.515-0.620)
17	1-methylurate	0.57	4.00E-03	0.653(0.558-0.749)	0.33	4.75E-16	0.683(0.634-0.732)
18	2-oxoarginine*	0.59	2.45E-07	0.770(0.695-0.845)	0.36	2.33E-14	0.709(0.661-0.758)
19	tiglylcarnitine (C5:1-DC)	0.60	5.35E-05	0.735(0.649-0.821)	0.58	1.78E-16	0.700(0.653-0.748)
20	androstenediol (3beta,17beta) monosulfate (1)	0.61	1.88E-04	0.686(0.596-0.775)	0.84	6.82E-03	0.559(0.507-0.612)
21	linoleoyl-arachidonoyl-glycerol (18:2/20:4) [2]*	0.61	3.18E-05	0.717(0.626-0.808)	0.78	6.66E-03	0.578(0.525-0.631)
22	2,3-dihydroxyisovalerate	0.62	1.60E-06	0.787(0.713-0.860)	0.46	1.36E-26	0.791(0.749-0.833)
23	isovalerylcarnitine (C5)	0.64	9.68E-09	0.757(0.680-0.833)	0.46	1.90E-38	0.847(0.812-0.883)
24	oleoyl-arachidonoyl-glycerol (18:1/20:4) [2]*	0.64	7.67E-05	0.722(0.631-0.813)	0.74	1.75E-06	0.613(0.561-0.665)
25	beta-alanine	0.65	6.78E-05	0.720(0.636-0.805)	0.94	2.32E-03	0.552(0.498-0.605)
26	3-methylhistidine	0.66	3.43E-03	0.667(0.579-0.756)	0.88	1.19E-02	0.548(0.495-0.601)

FC: fold change; FDR: false discovery rate; AUC: area under the curve; CI: confidence interval

Appendix Table S3. LRScores of 26 selected features.

Feature	Metabolites	LRScore
1	2,6-dihydroxybenzoic acid	1.72
2	cysteine s-sulfate	1.57
3	isovalerylcarnitine (C5)	1.50
4	glycoursodeoxycholate	1.09
5	2-hydroxy-3-methylvalerate	0.96
6	N-acetylneuraminate	0.87
7	oxindolylalanine	0.76
8	acisoga	0.69
9	glycochenodeoxycholate	0.60
10	maltose	0.43
11	hexanoylglutamine	0.30
12	beta-alanine	0.27
13	androstenediol (3beta,17beta) monosulfate (1)	0.19
14	3-hydroxybutyrate (BHBA)	0.17
15	1-methylurate	0.07
16	glycerol	0.01
17	3-methylhistidine	0.00
18	C-glycosyltryptophan	0.00
19	N6-succinyladenosine	0.00
20	2,3-dihydroxyisovalerate	0.00
21	pentose acid**	0.00
22	tiglylcarnitine (C5:1-DC)	0.00
23	2-oxoarginine*	0.00
24	acetoacetate	0.00
25	linoleoyl-arachidonoyl-glycerol (18:2/20:4) [2]*	0.00
26	oleoyl-arachidonoyl-glycerol (18:1/20:4) [2]*	0.00

LRScore: lasso regression score

Appendix Table S4. The diagnostic performance of 26 replicated blood metabolites in the discovery and validation dataset.

Machine learning	Performance	Discovery dataset	Validation dataset
NN	**Sensitivity (95%CI)**	0.900(0.774-0.963)	0.846(0.791-0.889)
	**Specificity (95%CI)**	0.904(0.822-0.953)	0.903(0.854-0.937)
	**Accuracy (95%CI)**	0.903(0.839-0.944)	0.874(0.839-0.903)
	**AUC (95%CI)**	0.957(0.928-0.987)	0.944(0.924-0.964)
SVM	**Sensitivity (95%CI)**	0.860(0.726-0.937)	0.811(0.752-0.858)
	**Specificity (95%CI)**	0.968(0.903-0.992)	0.876(0.824-0.915)
	**Accuracy (95%CI)**	0.931(0.873-0.964)	0.843(0.806-0.875)
	**AUC (95%CI)**	0.971(0.949-0.993)	0.908(0.882-0.935)
RR	**Sensitivity (95%CI)**	0.900(0.774-0.963)	0.881(0.830-0.919)
	**Specificity (95%CI)**	0.904(0.822-0.953)	0.810(0.751-0.858)
	**Accuracy (95%CI)**	0.903(0.839-0.944)	0.845(0.808-0.877)
	**AUC (95%CI)**	0.957(0.925-0.988)	0.916(0.891-0.941)
LR	**Sensitivity (95%CI)**	0.940(0.825-0.984)	0.863(0.810-0.904)
	**Specificity (95%CI)**	0.915(0.834-0.960)	0.885(0.834-0.922)
	**Accuracy (95%CI)**	0.924(0.864-0.959)	0.874(0.839-0.903)
	**AUC (95%CI)**	0.960(0.927-0.992)	0.933(0.911-0.955)
NB	**Sensitivity (95%CI)**	0.800(0.659-0.895)	0.903(0.855-0.937)
	**Specificity (95%CI)**	0.840(0.747-0.905)	0.894(0.844-0.929)
	**Accuracy (95%CI)**	0.826(0.752-0.882)	0.898(0.866-0.924)
	**AUC (95%CI)**	0.898(0.849-0.947)	0.950(0.931-0.969)

NN: neural network; SVM: support vector machine; RR: ridge regression; LR: lasso regression; NB: naive bayes; AUC: area under the curve; CI: confidence interval

Appendix Table S5. Average diagnostic scores of 26 metabolites in the discovery dataset and validation dataset.

**Algorithm**	**Dataset**		**Average score**		***P***
			**GC**	**Control**	
NN	Discovery dataset	Fold 1	0.866	0.076	< 0.001
		Fold 2	0.563	0.081	0.006
		Fold 3	0.843	0.056	< 0.001
		Fold 4	0.853	0.069	< 0.001
		Fold 5	0.854	0.150	< 0.001
	Verification dataset		0.682	0.068	< 0.001
SVM	Discovery dataset	Fold 1	0.843	0.096	< 0.001
		Fold 2	0.605	0.101	0.002
		Fold 3	0.866	0.060	< 0.001
		Fold 4	0.875	0.090	< 0.001
		Fold 5	0.822	0.130	< 0.001
	Verification dataset		0.694	0.156	< 0.001
RR	Discovery dataset	Fold 1	0.878	0.081	< 0.001
		Fold 2	0.546	0.077	0.007
		Fold 3	0.850	0.056	< 0.001
		Fold 4	0.896	0.073	< 0.001
		Fold 5	0.861	0.143	< 0.001
	Verification dataset		0.659	0.094	< 0.001
LR	Discovery dataset	Fold 1	0.892	0.082	< 0.001
		Fold 2	0.602	0.102	0.002
		Fold 3	0.873	0.065	< 0.001
		Fold 4	0.904	0.084	< 0.001
		Fold 5	0.760	0.152	< 0.001
	Verification dataset		0.724	0.095	< 0.001
NB	Discovery dataset	Fold 1	0.900	0.180	< 0.001
		Fold 2	0.417	0.087	0.063
		Fold 3	0.901	0.288	< 0.001
		Fold 4	0.934	0.163	< 0.001
		Fold 5	0.670	0.054	0.002
	Verification dataset		0.821	0.089	< 0.001

GC: gastric cancer; NN: neural network; SVM: support vector machine; RR: ridge regression; LR: lasso regression; NB: naive bayes

Appendix Table S6. Average diagnostic scores of six metabolic biomarkers in the discovery and validation dataset.

Algorithm	Dataset		Average score		*P*
			GC	Control	
NN	Discovery dataset	Fold 1	0.902	0.051	<0.001
		Fold 2	0.737	0.116	<0.001
		Fold 3	0.868	0.054	<0.001
		Fold 4	0.937	0.115	<0.001
		Fold 5	0.714	0.133	<0.001
	Verification dataset		0.784	0.101	<0.001
SVM	Discovery dataset	Fold 1	0.848	0.077	<0.001
		Fold 2	0.734	0.088	<0.001
		Fold 3	0.795	0.075	<0.001
		Fold 4	0.855	0.116	<0.001
		Fold 5	0.852	0.146	<0.001
	Verification dataset		0.740	0.152	<0.001
RR	Discovery dataset	Fold 1	0.857	0.056	<0.001
		Fold 2	0.722	0.119	<0.001
		Fold 3	0.833	0.062	<0.001
		Fold 4	0.914	0.127	<0.001
		Fold 5	0.697	0.125	<0.001
	Verification dataset		0.753	0.096	<0.001
LR	Discovery dataset	Fold 1	0.889	0.055	<0.001
		Fold 2	0.741	0.148	<0.001
		Fold 3	0.847	0.061	<0.001
		Fold 4	0.927	0.129	<0.001
		Fold 5	0.723	0.133	<0.001
	Verification dataset		0.779	0.098	<0.001
NB	Discovery dataset	Fold 1	0.828	0.134	<0.001
		Fold 2	0.635	0.138	<0.001
		Fold 3	0.812	0.145	<0.001
		Fold 4	0.866	0.168	<0.001
		Fold 5	0.583	0.113	0.004
	Verification dataset		0.739	0.148	<0.001

GC: gastric cancer; NN: neural network; SVM: support vector machine; RR: ridge regression; LR: lasso regression; NB: naive bayes

Appendix Table S7. The diagnostic sensitivity of metabolic biomarker panel compared with clinical protein biomarker tests.

	**Discovery dataset**		**Validation dataset**	
	**Sensitivity**	**95% CI**	**Sensitivity**	**95% CI**
Met-NN(6)	0.940	0.825-0.984	0.925	0.881-0.954
CA724	0.240	0.131-0.382	0.148	0.103-0.203
CA199	0.180	0.086-0.314	0.126	0.085-0.177
CA242	0.160	0.072-0.291	0.094	0.058-0.141
CEA	0.080	0.022-0.192	0.072	0.042-0.114
CA125	0.020	0.001-0.106	0.079	0.046-0.125
AFP	0.020	0.001-0.106	0.031	0.013-0.064

CA724: carbohydrate antigen 724; CA199: carbohydrate antigen 199; CA242: carbohydrate antigen 242; CA125: carbohydrate antigen 125; CEA: carcinoembryonic antigen; AFP: alpha-fetoprotein; CI: confidence interval

Appendix Table S8. The diagnostic performance of six metabolic biomarkers in the discovery and validation dataset, adjusting age, sex, smoking and drinking.

Machine learning	Performance	GC (all stage)		GC (Early stage)	
		Discovery dataset	Validation dataset	Discovery dataset	Validation dataset
NN	Sensitivity (95%CI)	0.940 (0.825-0.984)	0.877(0.825-0.915)	0.895(0.655-0.982)	0.887(0.811-0.936)
	Specificity (95%CI)	0.936 (0.861-0.974)	0.854(0.800-0.896)	0.947(0.875-0.980)	0.836(0.780-0.881)
	Accuracy (95%CI)	0.938 (0.881-0.969)	0.865(0.830-0.895)	0.938(0.872-0.973)	0.853(0.810-0.888)
	AUC (95%CI)	0.980(0.963-0.997)	0.928(0.904-0.952)	0.973(0.943-1.002)	0.910(0.876-0.943)
SVM	Sensitivity (95%CI)	0.920 (0.799-0.974)	0.890(0.840-0.926)	0.947(0.719-0.997)	0.843(0.761-0.902)
	Specificity (95%CI)	0.957 (0.888-0.986)	0.850(0.795-0.892)	0.894(0.809-0.945)	0.810(0.751-0.858)
	Accuracy (95%CI)	0.944 (0.890-0.974)	0.870(0.834-0.899)	0.903(0.829-0.948)	0.821(0.775-0.859)
	AUC (95%CI)	0.975(0.955-0.995)	0.921(0.896-0.946)	0.953(0.915-0.991)	0.887(0.848-0.926)
RR	Sensitivity (95%CI)	0.940 (0.825-0.984)	0.934(0.891-0.961)	0.895(0.655-0.982)	0.835(0.751-0.895)
	Specificity (95%CI)	0.904 (0.822-0.953)	0.814(0.756-0.861)	0.968(0.903-0.992)	0.925(0.880-0.954)
	Accuracy (95%CI)	0.917 (0.856-0.954)	0.874(0.839-0.903)	0.956(0.895-0.984)	0.894(0.856-0.924)
	AUC (95%CI)	0.975(0.954-0.995)	0.935(0.912-0.958)	0.954(0.917-0.992)	0.932(0.905-0.959)
LR	Sensitivity (95%CI)	0.940 (0.825-0.984)	0.899(0.850-0.933)	0.789(0.539-0.930)	0.843(0.761-0.902)
	Specificity (95%CI)	0.904 (0.822-0.953)	0.876(0.824-0.915)	0.979(0.918-0.996)	0.889(0.839-0.926)
	Accuracy (95%CI)	0.917 (0.856-0.954)	0.887(0.854-0.914)	0.947(0.883-0.978)	0.874(0.833-0.906)
	AUC (95%CI)	0.969(0.946-0.993)	0.940(0.918-0.962)	0.929(0.881-0.978)	0.922(0.892-0.951)
NB	Sensitivity (95%CI)	0.800 (0.659-0.895)	0.806(0.747-0.854)	0.895(0.655-0.982)	0.809(0.723-0.874)
	Specificity (95%CI)	0.979 (0.918-0.996)	0.872(0.819-0.911)	0.862(0.772-0.921)	0.854(0.800-0.896)
	Accuracy (95%CI)	0.917 (0.856-0.954)	0.839(0.801-0.871)	0.867(0.787-0.921)	0.839(0.794-0.875)
	AUC (95%CI)	0.943(0.910-0.977)	0.922(0.898-0.946)	0.915(0.860-0.971)	0.907(0.876-0.938)

GC: gastric cancer; NN: neural network; SVM: support vector machine; RR: ridge regression; LR: lasso regression; NB: naive bayes; AUC: area under the curve; CI: confidence interval

Appendix Table S9. Details of the genome-wide association studies and datasets used in MR analyses.

Exposure or outcome	Sample size	Ancestry	Links for data download	PMID
1,400 blood metabolites	8,299 participants	European ancestry		36635386
Malignant neoplasm of stomach	1,307 cases, 287,137 controls	European ancestry (Finngen R9)	https://storage.googleapis.com/finngen-public-data-r9/summary_stats/finngen_R9_C3_STOMACH_EXALLC.gz	/

Appendix Table S10. Characteristics of the genetic variants associated with the circulating level of isovalerylcarnitine (C5).

SNP	Trait	*P*	Effect_ allele.exposure	Other_ allele.exposure	eaf.exposure	beta.exposure	SE.exposure
rs10520079	GCST90199747	2.00E-06	A	G	0.337	-0.075	0.016
rs11152173	GCST90199747	7.50E-06	T	C	0.349	-0.071	0.016
rs1171616	GCST90199747	2.26E-08	T	G	0.770	0.101	0.018
rs117747447	GCST90199747	7.49E-06	C	G	0.023	-0.219	0.049
rs117793326	GCST90199747	9.32E-06	G	A	0.014	0.284	0.064
rs117855554	GCST90199747	7.64E-06	T	G	0.041	-0.166	0.037
rs118170877	GCST90199747	1.00E-06	G	A	0.015	0.305	0.062
rs12112191	GCST90199747	8.70E-06	G	A	0.883	0.103	0.023
rs140582732	GCST90199747	8.56E-06	T	C	0.018	-0.251	0.056
rs143854855	GCST90199747	8.13E-06	T	A	0.024	-0.214	0.048
rs144720400	GCST90199747	8.27E-06	T	C	0.009	0.344	0.077
rs149719179	GCST90199747	6.81E-06	C	A	0.016	0.271	0.060
rs152815	GCST90199747	3.20E-10	A	G	0.387	-0.097	0.015
rs1801591	GCST90199747	5.69E-06	A	G	0.087	0.119	0.026
rs192431765	GCST90199747	8.83E-08	C	G	0.008	0.450	0.084
rs2289329	GCST90199747	2.93E-29	C	T	0.384	-0.170	0.015
rs273900	GCST90199747	1.89E-32	A	G	0.620	-0.180	0.015
rs34708625	GCST90199747	8.76E-10	C	T	0.265	0.103	0.017
rs36113945	GCST90199747	2.65E-06	C	A	0.175	-0.092	0.020
rs4277318	GCST90199747	1.60E-07	G	A	0.782	-0.097	0.018
rs4666393	GCST90199747	7.66E-06	C	T	0.243	-0.078	0.017
rs4705885	GCST90199747	5.33E-09	A	G	0.372	-0.090	0.015
rs55665473	GCST90199747	5.33E-06	A	G	0.245	-0.078	0.017
rs62525488	GCST90199747	3.21E-06	G	A	0.032	0.194	0.042
rs6749227	GCST90199747	2.13E-06	C	T	0.086	0.127	0.027
rs74759468	GCST90199747	6.70E-06	T	C	0.015	0.275	0.061
rs74860073	GCST90199747	4.30E-07	A	G	0.009	-0.389	0.077
rs7707728	GCST90199747	2.33E-07	T	C	0.118	0.121	0.023
rs79211833	GCST90199747	9.88E-06	C	T	0.097	-0.110	0.025
rs80329585	GCST90199747	1.27E-06	C	A	0.011	-0.345	0.071
rs9976940	GCST90199747	7.83E-06	G	C	0.048	-0.155	0.035

Appendix Table S11. N-acetylneuraminate showed no statistically significant association with GC in Mendelian randomization analyses.

**N-acetylneuraminate**	**SNP**	**Beta**	**SE**	***P***	**Hets**	**Pleios**
Inverse variance weighted (fixed effects)	28	0.020	0.087	0.819	0.808	
Maximum likelihood	28	0.021	0.088	0.815		
Simple median	28	0.069	0.141	0.624		
Weighted median	28	-0.026	0.128	0.840		
MR Egger	28	-0.014	0.157	0.929		0.797

GC: gastric cancer; SNP: single nucleotide polymorphism；SE：standard error

Appendix Table S12. Genetic variants associated with the circulating level of isovalerylcarnitine (C5) in reverse Mendelian randomization analyses.

**Methods**	**SNPs**	**Beta**	**SE**	**OR**	**95%Cl**	***P***	**Qrs**
Inverse-variance weighted (random effects)	14	0.021	0.019	1.022	0.985-1.060	0.256	0.805
Inverse-variance weighted (fixed effects)	14	0.021	0.019	1.022	0.985-1.060	0.256	0.805
MR-Egger	14	-0.018	0.048	0.982	0.894-1.078	0.703	NA
Weighted median	14	0.015	0.026	1.015	0.966-1.067	0.559	NA
Simple median	14	0.010	0.026	1.010	0.960-1.063	0.692	NA
Maximum-likelihood method	14	0.022	0.019	1.022	0.984-1.061	0.255	NA

SNP: single nucleotide polymorphism；SE：standard error

Appendix Table S13. Differentially expressed proteins significantly correlated with isovalerylcarnitine (C5) expression.

Protein	r	*p.value*	Protein	r	*p.value*	Protein	r	*p.value*	Protein	r	*p.value*
IGHV1.24	0.167	4.52E-02	KRT10	-0.256	1.92E-03	CD81	0.18	3.11E-02	DENND6A	-0.226	6.41E-03
ARHGAP10	0.172	3.98E-02	KRT13	-0.233	4.94E-03	TPI1	-0.246	2.90E-03	DDX60	0.165	4.80E-02
SSC5D	-0.321	8.66E-05	KRT5	-0.249	2.65E-03	PSMA6	-0.209	1.18E-02	LRRC47	0.167	4.48E-02
TTC24	0.319	9.65E-05	ACP5	0.185	2.61E-02	S100A10	-0.233	5.03E-03	KRT78	-0.258	1.82E-03
WDR91	-0.197	1.77E-02	APEH	-0.228	6.09E-03	LYZ	-0.288	4.64E-04	BPIFB2	-0.201	1.58E-02
CNOT1	-0.176	3.48E-02	GYS1	0.191	2.21E-02	VBP1	0.168	4.47E-02	OXR1	0.215	9.74E-03
SMCHD1	0.179	3.15E-02	ENO3	0.223	7.09E-03	NPC2	-0.201	1.58E-02	CERS5	-0.287	4.83E-04
TMEM212	0.187	2.47E-02	SELL	0.21	1.14E-02	RPS16	-0.218	8.75E-03	JAGN1	0.292	3.90E-04
A2ML1	-0.27	1.05E-03	CD99	0.172	3.98E-02	RPS18	-0.22	7.97E-03	LINC02694	-0.188	2.42E-02
PSMD11	-0.185	2.63E-02	CPM	-0.307	1.80E-04	RPS28	-0.165	4.85E-02	PCSK9	-0.312	1.44E-04
QSOX1	-0.447	1.89E-08	IDE	-0.228	5.91E-03	RPL31	0.201	1.56E-02	MTMR14	-0.205	1.36E-02
CHL1	-0.175	3.62E-02	JUP	-0.294	3.53E-04	HBG2	-0.249	2.64E-03	LFNG	-0.299	2.76E-04
CCL21	0.27	1.09E-03	GLUL	-0.309	1.66E-04	KRT83	-0.243	3.30E-03	CHST13	0.215	9.52E-03
ISLR	-0.201	1.57E-02	B4GALT1	-0.22	8.15E-03	RELN	-0.24	3.79E-03	APPL2	0.206	1.31E-02
ABLIM1	-0.22	8.10E-03	ACP3	-0.411	3.10E-07	SRPX	-0.18	3.13E-02	SDR9C7	-0.275	8.35E-04
IMPA2	-0.211	1.12E-02	ELN	-0.476	1.61E-09	GPLD1	-0.181	2.97E-02	OR4D6	-0.241	3.64E-03
SLC9A3R1	0.196	1.85E-02	ST6GAL1	0.284	5.63E-04	NUCB2	-0.188	2.42E-02	PRSS3P2	-0.32	9.15E-05
PSMA7	-0.219	8.44E-03	DSP	-0.256	1.93E-03	DCD	-0.226	6.42E-03	FAM76A	-0.334	4.29E-05
LECT2	0.228	6.08E-03	TIMP2	-0.322	8.34E-05	TNFAIP6	-0.5	1.73E-10	ITFG1	-0.184	2.69E-02
MCF2L	0.166	4.73E-02	CBR1	-0.178	3.27E-02	HSPG2	-0.445	2.30E-08	PREX1	-0.195	1.93E-02
ADAMDEC1	0.166	4.67E-02	ANK1	-0.21	1.14E-02	SORD	-0.312	1.41E-04	AGR3	-0.17	4.16E-02
CHAD	0.167	4.52E-02	NAGA	-0.189	2.34E-02	CAP1	0.175	3.61E-02	BPIFB1	-0.256	1.98E-03
CCL16	0.174	3.69E-02	CAPN2	-0.208	1.24E-02	KRT76	-0.263	1.45E-03	SNX29	0.177	3.37E-02
PHGDH	-0.214	9.97E-03	LGALS3	-0.234	4.70E-03	RHAG	-0.238	4.11E-03	SNED1	-0.37	4.87E-06
KLK10	-0.164	4.89E-02	IGFBP3	0.206	1.34E-02	CYP27A1	0.263	1.42E-03	POF1B	-0.279	7.03E-04
TGM5	-0.205	1.36E-02	IGFBP2	-0.307	1.84E-04	DSG1	-0.279	7.00E-04	ITLN1	-0.201	1.55E-02
ENSA	-0.188	2.38E-02	ERCC2	0.194	2.00E-02	DSC2	-0.385	1.92E-06	OIT3	-0.326	6.53E-05
MGRN1	0.204	1.44E-02	LBP	-0.32	9.36E-05	NUCB1	-0.242	3.48E-03	TTN	0.202	1.54E-02
AQR	-0.438	4.11E-08	CDH2	-0.407	4.08E-07	ITIH3	-0.428	8.84E-08	PSMF1	-0.248	2.69E-03
PIP5K1C	-0.413	2.70E-07	VCAM1	-0.33	5.47E-05	PTPN11	0.207	1.27E-02	NRGN	0.214	1.01E-02
PPL	-0.166	4.73E-02	PI3	-0.293	3.67E-04	PSME1	-0.192	2.13E-02	FRZB	0.242	3.53E-03
NRP2	-0.252	2.29E-03	TYMP	-0.228	5.90E-03	CKAP4	-0.245	3.12E-03	EVPL	-0.201	1.57E-02
CTSV	-0.237	4.20E-03	ANXA7	-0.302	2.36E-04	LRP1	-0.312	1.42E-04	GGH	-0.262	1.53E-03
PFDN1	0.179	3.15E-02	PSMB1	-0.214	9.85E-03	PCDH1	-0.436	4.56E-08	KHSRP	-0.383	2.18E-06
TMCC2	0.246	2.91E-03	PZP	-0.319	9.49E-05	TGM3	-0.244	3.21E-03	PRG4	0.343	2.60E-05
CPNE3	-0.239	3.98E-03	OGN	-0.234	4.79E-03	LGALS3BP	-0.212	1.09E-02	HGD	0.196	1.83E-02
RAB11FIP3	0.232	5.09E-03	C4BPB	-0.379	2.85E-06	MFGE8	0.337	3.56E-05	MAL2	-0.26	1.65E-03
GGCT	-0.228	6.07E-03	FLG	-0.294	3.48E-04	DSC1	-0.278	7.41E-04	NCLN	0.189	2.31E-02
PDCD6	-0.346	2.16E-05	RASA1	0.215	9.72E-03	FGL1	-0.233	4.90E-03	PBXIP1	0.235	4.61E-03
ALOX12B	-0.277	7.60E-04	CSRP1	0.189	2.30E-02	ASPH	0.223	7.12E-03	AIDA	0.174	3.73E-02
VPS26A	-0.218	8.73E-03	NF1	0.219	8.37E-03	EFEMP1	-0.533	6.03E-12	FOXRED1	0.238	4.11E-03
CREG1	-0.218	8.74E-03	UBA1	0.264	1.37E-03	SF3A3	-0.187	2.51E-02	ZG16B	-0.176	3.54E-02
SERPINB7	-0.257	1.89E-03	GPX3	-0.234	4.74E-03	IRAG2	0.197	1.81E-02	BPIFA2	-0.183	2.83E-02
FCN3	0.192	2.12E-02	SPRR2D	-0.274	9.09E-04	MYO1E	-0.186	2.55E-02	CYFIP2	-0.182	2.93E-02
ATP6AP2	-0.383	2.21E-06	FDXR	-0.164	4.95E-02	SPP2	-0.398	7.66E-07	CPNE2	-0.18	3.12E-02
IDH1	-0.213	1.03E-02	TGM1	-0.26	1.68E-03	PSMD2	-0.251	2.42E-03	S100A16	-0.303	2.20E-04
ANXA9	-0.179	3.17E-02	CPN2	-0.351	1.61E-05	DNAJC3	0.438	3.93E-08	PERP	-0.327	6.21E-05
CCN5	-0.346	2.18E-05	CES1	0.276	8.14E-04	SELENBP1	-0.18	3.06E-02	MRPL48	0.167	4.54E-02
MYO1D	-0.262	1.50E-03	FBLN1	-0.395	9.90E-07	EIF3I	-0.175	3.57E-02	ZC3HAV1L	0.203	1.48E-02
NDUFA3	0.197	1.81E-02	SFPQ	-0.282	6.21E-04	TARBP1	-0.19	2.24E-02	CRELD1	0.192	2.11E-02
GOSR1	0.169	4.30E-02	CA6	-0.192	2.10E-02	MSLN	-0.231	5.34E-03	SFRP2	0.363	7.94E-06
TNFAIP8	0.166	4.70E-02	WARS1	-0.213	1.05E-02	MADCAM1	0.184	2.70E-02	REEP6	-0.287	4.95E-04
PAPLN	-0.444	2.44E-08	PTPRB	0.273	9.22E-04	ASAH1	-0.319	9.74E-05	LRCH3	-0.186	2.56E-02
APOM	0.193	2.06E-02	PTPRG	0.185	2.66E-02	RIPK1	-0.203	1.45E-02	SEC22A	-0.181	2.97E-02
VNN1	-0.178	3.32E-02	LORICRIN	-0.206	1.32E-02	DNASE1L3	0.204	1.42E-02	COG3	0.236	4.49E-03
EFEMP2	-0.46	6.74E-09	AHCY	-0.222	7.38E-03	ITGA7	-0.25	2.47E-03	ZNF462	-0.325	7.04E-05
SCGB1D2	-0.312	1.37E-04	BDNF	0.204	1.41E-02	PKP1	-0.238	4.14E-03	DCHS1	-0.381	2.51E-06
RECK	-0.182	2.92E-02	F8A1	0.26	1.62E-03	BLMH	-0.277	7.70E-04	PDLIM2	-0.218	8.72E-03
CYB5A	-0.262	1.51E-03	IGFBP5	0.281	6.38E-04	SNTB1	0.175	3.64E-02	SUSD3	0.175	3.61E-02
F8	-0.322	8.44E-05	ACP1	-0.207	1.27E-02	DSG2	-0.473	2.13E-09	PRRC1	-0.27	1.05E-03
PNP	-0.299	2.79E-04	POLR2A	-0.195	1.93E-02	EBI3	-0.423	1.27E-07	KLHL15	-0.28	6.83E-04
HPRT1	0.175	3.58E-02	PSMA1	-0.307	1.80E-04	FGL2	-0.177	3.34E-02	FAM20A	0.174	3.66E-02
C1R	-0.382	2.35E-06	PSMA2	-0.301	2.50E-04	FLNC	0.173	3.79E-02	FAM161B	-0.288	4.69E-04
CFD	0.17	4.13E-02	PSMA3	-0.216	9.20E-03	GAS6	-0.167	4.53E-02	SERPINB12	-0.307	1.85E-04
PDGFB	0.249	2.65E-03	PSMA4	-0.189	2.36E-02	RBM39	0.203	1.47E-02	PGLYRP2	-0.532	6.47E-12
IGHV1.46	0.165	4.88E-02	MGAT1	-0.195	1.91E-02	HABP2	0.213	1.03E-02	GSDMA	-0.268	1.15E-03
PIGR	-0.199	1.71E-02	PON1	-0.211	1.13E-02	IHH	0.228	5.96E-03	HS3ST6_HS3ST4_HS3ST3	0.169	4.29E-02

									B1_HS3ST3A1		
COL3A1	-0.189	2.29E-02	MAOB	-0.195	1.90E-02	INPP5A	0.172	3.88E-02	SPRR2F	-0.194	1.96E-02
KRT14	-0.214	1.02E-02	YWHAQ	0.193	2.03E-02	RFTN1	-0.18	3.04E-02	PEBP4	0.187	2.51E-02
KRT6A	-0.183	2.78E-02	CFP	0.27	1.04E-03	PPP2R5D	0.178	3.25E-02	KRT12	-0.234	4.68E-03
SPTA1	-0.203	1.45E-02	AOAH	-0.312	1.41E-04	LTBP2	-0.277	7.86E-04	PSMD1	-0.222	7.45E-03
APOE	0.196	1.88E-02	PSMB8	0.204	1.44E-02	GPNMB	-0.267	1.21E-03	PFDN5	0.246	2.92E-03
APOC1	-0.232	5.06E-03	PSMA5	-0.257	1.90E-03	KRT72	-0.174	3.68E-02	VAT1	-0.358	1.04E-05
MBP	-0.285	5.23E-04	PSMB6	-0.212	1.09E-02	PSMD6	-0.225	6.67E-03	MMP19	-0.415	2.37E-07
SLC4A1	-0.197	1.78E-02	PSMB5	-0.167	4.50E-02	POSTN	-0.247	2.90E-03	FEZ1	-0.181	3.03E-02
APCS	0.188	2.41E-02	TMOD1	-0.218	8.55E-03	PON3	-0.282	6.04E-04	RARRES2	0.179	3.19E-02
C9	-0.367	6.20E-06	CCN2	0.227	6.11E-03	RCN1	-0.242	3.43E-03	TREX2	-0.306	1.91E-04
APOH	0.221	7.83E-03	CRABP2	-0.259	1.72E-03	SHH	0.216	9.45E-03	WDR77	-0.165	4.85E-02
FN1	-0.496	2.64E-10	SERPINB3	-0.224	7.00E-03	FCN2	0.258	1.78E-03	DESI2	0.164	4.97E-02
AHSG	-0.189	2.36E-02	MARCKS	0.239	3.97E-03	CDSN	-0.287	4.86E-04	DCTN5	0.323	7.72E-05
TFRC	-0.252	2.32E-03	BLVRB	-0.314	1.24E-04	TRIP10	0.232	5.12E-03	MYL10	0.164	4.92E-02
HPX	-0.299	2.67E-04	PEBP1	-0.181	3.01E-02	MYLK	0.188	2.38E-02	SPON2	-0.177	3.43E-02
F11	0.184	2.69E-02	CCND3	0.164	4.98E-02	ATP6AP1	-0.344	2.38E-05	MENT	-0.181	2.95E-02
MMP1	-0.165	4.83E-02	SRI	-0.226	6.41E-03	BCL2A1	0.309	1.61E-04	CHID1	0.414	2.53E-07
SLPI	0.2	1.61E-02	S100A7	-0.299	2.67E-04	DPYSL2	0.214	9.96E-03	B3GNT5	0.215	9.75E-03
C4BPA	-0.398	7.81E-07	SLC6A4	0.184	2.76E-02	ECM1	-0.342	2.67E-05	FGFBP2	0.261	1.61E-03
CAT	-0.251	2.43E-03	CASP14	-0.215	9.64E-03	CYP1B1	-0.429	8.02E-08	ALOXE3	-0.29	4.16E-04
GBA	-0.33	5.44E-05	SFN	-0.2	1.63E-02	CLPP	0.223	7.19E-03	UACA	0.24	3.75E-03
FUCA1	-0.169	4.25E-02	S100A11	-0.182	2.94E-02	QPCT	-0.175	3.57E-02	KRT23	-0.199	1.70E-02
HRG	-0.18	3.10E-02	PRDX2	-0.283	5.86E-04	PTPRO	-0.314	1.28E-04	PDGFD	0.206	1.34E-02
THY1	-0.348	1.89E-05	CDA	-0.193	2.08E-02	KRT24	-0.181	3.01E-02	CRISPLD2	0.335	4.12E-05
KRT1	-0.281	6.36E-04	CDH5	-0.445	2.24E-08	FER1L6	-0.188	2.39E-02	ARL6	-0.196	1.86E-02
VWF	-0.298	2.87E-04	MAN1A1	-0.235	4.51E-03	ADPRM	-0.362	8.17E-06	C11orf54	-0.218	8.62E-03
SHBG	-0.501	1.66E-10	KDELR2	-0.18	3.09E-02	PLA2G4E	-0.179	3.14E-02	ANAPC1	0.17	4.18E-02
ASL	0.279	7.11E-04	PRSS3	0.19	2.26E-02	ZIK1	-0.289	4.53E-04	RNASE7	-0.354	1.38E-05
HSPB1	-0.32	9.08E-05	CTNNA1	-0.187	2.50E-02	KRT71	-0.379	2.83E-06	SLK	0.171	4.05E-02
CYBB	-0.18	3.10E-02	SPRR2A	-0.236	4.44E-03	PARP14	0.179	3.18E-02	PNN	-0.225	6.58E-03
IGF1	0.241	3.63E-03	THBS2	-0.217	9.04E-03	CERKL	0.239	3.98E-03	DPAGT1	-0.171	4.04E-02
ARG1	-0.301	2.41E-04	KRT9	-0.275	8.57E-04	ERFE	-0.188	2.37E-02	RNPEP	-0.259	1.72E-03
S100A8	-0.272	9.55E-04	SAA4	0.259	1.70E-03	ACSF3	0.191	2.15E-02	SMOC1	-0.23	5.46E-03
SERPINE1	0.192	2.10E-02	FBN1	-0.468	3.29E-09	SVEP1	-0.505	1.04E-10	HEATR1	0.169	4.23E-02
SERPINA5	0.295	3.30E-04	GLRX	-0.264	1.37E-03	TP53I3	-0.166	4.63E-02	TUBA4B	0.18	3.08E-02
SERPING1	-0.256	1.98E-03	KRT2	-0.29	4.31E-04	FBLN7	-0.182	2.89E-02	NT5DC2	0.196	1.85E-02
ALPL	-0.282	6.30E-04	SERPINB5	-0.17	4.17E-02	ASPRV1	-0.212	1.06E-02	RMI1	-0.164	4.93E-02
HMGN2_H MGN3	-0.199	1.69E-02	GPX4	0.217	9.11E-03	RUNDC3A	0.209	1.21E-02	COPS7B	0.176	3.52E-02
RPLP2	-0.175	3.59E-02	CAPG	-0.295	3.27E-04	FLG2	-0.256	1.95E-03	SCPEP1	-0.231	5.24E-03
RPLP0	-0.183	2.85E-02	MPL	0.183	2.77E-02	HERC4	0.183	2.80E-02	CDHR5	-0.431	6.94E-08
HPN	-0.195	1.89E-02	PSMB10	-0.218	8.54E-03	SLC35D3	0.191	2.15E-02	MUC5B	-0.202	1.52E-02
GLA	-0.272	9.73E-04	ADH7	-0.287	4.78E-04	KPRP	-0.338	3.39E-05	CHD8	0.218	8.74E-03
S100A9	-0.261	1.55E-03	MDH1	-0.169	4.33E-02	XP32	-0.319	9.92E-05	S100A14	-0.269	1.11E-03
CKM	-0.191	2.15E-02	UBA7	0.217	8.95E-03	DDI2	-0.212	1.07E-02	RETN	-0.268	1.17E-03
NPM1	-0.213	1.03E-02	PPP1R2_ PPP1R2B	0.24	3.71E-03	SIRPB1	-0.167	4.55E-02	PCDH12	-0.476	1.71E-09
CRH	-0.218	8.60E-03	HAL	-0.252	2.31E-03	EMC10	0.202	1.53E-02	GPR108	0.168	4.40E-02
NEFL	-0.17	4.12E-02	RPL35	-0.222	7.36E-03	YOD1	-0.291	4.09E-04	SPINK5	0.233	5.04E-03
PROS1	-0.436	4.60E-08	PRCP	-0.267	1.23E-03	ZDHHC20	0.216	9.36E-03	CRTAC1	-0.277	7.93E-04
ASGR2	-0.438	4.14E-08	HTT	-0.175	3.58E-02	RAP1GAP2	0.296	3.18E-04	PRTFDC1	0.17	4.19E-02
CTSD	-0.265	1.32E-03	ALDH3B1	-0.218	8.76E-03	HBM	-0.307	1.85E-04	PLSCR4	0.203	1.48E-02
ANXA2	-0.231	5.26E-03	RPL5	-0.166	4.62E-02	KRT80	-0.238	4.13E-03	OTULINL	0.179	3.16E-02
IVL	-0.207	1.30E-02	RPS5	-0.191	2.19E-02	PSAPL1	-0.266	1.25E-03	TECR	-0.17	4.21E-02
PRSS2	0.236	4.33E-03	LGALS7	-0.231	5.25E-03	MZT2B	0.211	1.13E-02	GGA3	0.201	1.57E-02
HEXB	-0.204	1.40E-02	ME1	-0.309	1.65E-04	CAVIN1	-0.259	1.73E-03	IL37	-0.239	3.98E-03
CTSL	-0.178	3.23E-02	SERPINB4	-0.24	3.70E-03	PDXDC2P	-0.179	3.22E-02	IL36G	-0.165	4.85E-02
APRT	-0.258	1.83E-03	GSS	-0.303	2.27E-04	PLBD1	-0.198	1.75E-02	ADA2	-0.303	2.25E-04
CTSB	-0.455	1.01E-08	PIK3CG	0.173	3.77E-02	LRRC8B	-0.203	1.48E-02	C1RL	-0.17	4.22E-02
UQCRH	0.187	2.50E-02	MASP1	-0.308	1.72E-04	ADGRD1	0.267	1.20E-03	KLK14	-0.247	2.87E-03
TPM2	-0.178	3.32E-02	ADGRE5	-0.297	2.97E-04	CYP20A1	0.165	4.79E-02	TOMM7	0.172	3.87E-02
SLC3A2	-0.175	3.58E-02	ALDH9A1	-0.261	1.60E-03	GPR15L	0.29	4.22E-04	ABRACL	0.189	2.34E-02
MMP2	-0.419	1.68E-07	PPM1F	-0.171	4.02E-02	SBSN	-0.287	4.89E-04	VPS54	0.164	4.93E-02
MMP3	-0.189	2.31E-02	CASP4	-0.188	2.41E-02	OLFML1	0.182	2.89E-02	PEF1	-0.218	8.76E-03
PLEK	0.166	4.65E-02	PSMB2	-0.305	2.05E-04	ANGPTL8	0.298	2.93E-04	CTSF	-0.322	8.42E-05
CD14	-0.506	9.82E-11	RGS6	0.195	1.92E-02	PAMR1	0.19	2.22E-02	FBLN5	-0.481	1.03E-09
COL4A2	-0.348	1.87E-05	EIF2B2	-0.412	2.99E-07	VIT	0.262	1.54E-03	KLK11	-0.371	4.78E-06
HCK	-0.255	2.07E-03	GSK3A	0.194	1.99E-02	ADAMTSL4	-0.398	7.98E-07	MARCO	-0.336	3.87E-05
SERPINF2	-0.208	1.23E-02	SELENOP	0.351	1.59E-05	NAPRT	0.169	4.30E-02	CRCT1	-0.291	4.11E-04
KRT7	-0.272	9.58E-04	SULT1A1	0.211	1.11E-02	ZNF841	0.169	4.27E-02	SRP68	-0.213	1.02E-02

RPSA	-0.19	2.26E-02	CRIP1	-0.312	1.42E-04	BNC2	-0.178	3.30E-02	TAC3	-0.214	1.00E-02
ENO2	0.208	1.24E-02	SERPINB9	0.166	4.69E-02	ACP7	-0.378	3.01E-06	PCYOX1	0.25	2.52E-03
MMP7	-0.167	4.59E-02	ST13	-0.256	1.92E-03	NCCRP1	-0.339	3.28E-05	PFDN2	0.178	3.23E-02
LGALS1	-0.37	4.87E-06	PPT1	-0.303	2.18E-04	SMIM5	0.198	1.76E-02	PUF60	-0.305	2.00E-04
C1S	-0.296	3.21E-04	RENBP	0.179	3.15E-02	ADAMTS13	-0.441	3.24E-08	CPA4	-0.203	1.46E-02
POTEI	-0.221	7.66E-03	PSMD7	-0.219	8.24E-03	GDPD3	-0.233	4.99E-03	SCOC	0.209	1.20E-02
TRAPPC2_ TRAPPC2B	0.194	1.99E-02	SGSH	-0.251	2.44E-03	COPS6	0.193	2.06E-02	SERPINB13	-0.199	1.67E-02
AMY1B_AMY 1C_AMY1A	-0.222	7.37E-03	LUM	-0.343	2.52E-05	CHMP1B	0.205	1.35E-02	PLXNA1	-0.225	6.68E-03
GAA	-0.286	5.12E-04	PRELP	-0.352	1.49E-05	UBE3B	0.2	1.61E-02	GNPTG	-0.208	1.23E-02
TXN	-0.247	2.89E-03	HNRNPM	0.184	2.71E-02	KRT27	-0.242	3.54E-03	SERPINA10	-0.389	1.48E-06
CTSA	-0.255	2.01E-03	ZNF132	0.167	4.54E-02	C6orf120	0.24	3.82E-03	PCOLCE2	0.246	2.97E-03
TFPI	0.225	6.68E-03	CRIP2	-0.208	1.22E-02	MDGA2	0.228	5.99E-03	NOTCH3	-0.409	3.69E-07
PF4V1	0.172	3.97E-02	SUCLG1	0.202	1.52E-02	KRT77	-0.236	4.46E-03	KMT2B	0.168	4.37E-02
CLU	-0.246	2.96E-03	CTSC	-0.263	1.48E-03	ADAMTSL2	-0.423	1.33E-07	MAGED2	0.187	2.51E-02
MAP2	-0.181	2.96E-02	SUB1	-0.171	4.08E-02	TADA2B	-0.249	2.66E-03	MINPP1	-0.322	8.38E-05
LIPC	0.27	1.07E-03	USP14	-0.211	1.12E-02	ATG4D	0.165	4.81E-02	TFR2	-0.421	1.46E-07
SLC2A1	-0.187	2.49E-02	HSPA2	-0.282	6.18E-04	OAF	-0.237	4.28E-03	THSD7A	0.269	1.10E-03
SPTB	-0.188	2.44E-02	GALC	-0.269	1.11E-03	EVC2	-0.452	1.27E-08	JAG2	-0.384	2.08E-06
CETP	-0.312	1.41E-04	APOC4	0.257	1.89E-03	VPS36	0.247	2.81E-03	CLEC11A	-0.206	1.32E-02
PABPC1	-0.247	2.88E-03	PLTP	-0.462	5.80E-09	LSR	-0.298	2.87E-04	HIGD1A	0.251	2.41E-03
F5	0.246	2.90E-03	INHBC	-0.187	2.51E-02	CARM1	-0.174	3.67E-02	ITM2B	0.183	2.83E-02
IMPDH2	-0.262	1.52E-03	LAMB2	-0.215	9.69E-03	KRT73	-0.198	1.71E-02	SBNO2	-0.23	5.52E-03
PIP	-0.238	4.06E-03	CDH6	-0.332	4.87E-05	STX12	-0.19	2.22E-02	MEMO1	-0.251	2.44E-03
CKB	-0.186	2.54E-02	CDH11	-0.512	5.66E-11	CPNE8	-0.327	6.21E-05	FBXO7	-0.285	5.34E-04
CKMT1A	-0.221	7.73E-03	CDH13	-0.402	6.05E-07	KRTAP13.1	-0.186	2.53E-02	NEU2	-0.271	1.01E-03
MYL4	-0.255	2.03E-03	NPEPPS	-0.221	7.72E-03	PLD3	-0.305	2.05E-04	ANGPTL3	-0.39	1.36E-06
BMP1	-0.187	2.49E-02	BID	0.176	3.53E-02	KSR1	0.227	6.18E-03	RFNG	-0.476	1.68E-09
VCAN	-0.567	1.31E-13	CARD18	-0.238	4.04E-03	SERPINA12	-0.23	5.49E-03	CPQ	-0.375	3.64E-06
EEF2	-0.216	9.16E-03	MTPN	0.188	2.39E-02	WDFY1	0.179	3.19E-02	LHFPL6	-0.185	2.61E-02

Appendix Table S14. Sensitivity analysis on AUC across different missing value imputation methods.

	**Mean imputation**		**Median imputation**		**K-nearest neighbor (KNN) imputation**		**Probabilistic PCA imputation**	
	Discovery dataset	Validation dataset	Discovery dataset	Validation dataset	Discovery dataset	Validation dataset	Discovery dataset	Validation dataset
NN	0.964 (0.937-0.991)	0.937 (0.914-0.959)	0.965 (0.940-0.991)	0.939 (0.916-0.961)	0.971 (0.949-0.994)	0.940 (0.918-0.962)	0.973 (0.953-0.994)	0.943 (0.922-0.963)
SVM	0.950 (0.916-0.983)	0.906 (0.879-0.934)	0.949 (0.914-0.983)	0.908 (0.880-0.935)	0.952 (0.920-0.983)	0.909 (0.882-0.936)	0.963 (0.936-0.990)	0.913 (0.887-0.940)
RR	0.962 (0.935-0.990)	0.937 (0.914-0.959)	0.966 (0.940-0.991)	0.939 (0.917-0.961)	0.972 (0.950-0.994)	0.939 (0.917-0.961)	0.971 (0.948-0.994)	0.941 (0.919-0.962)
LR	0.962 (0.934-0.990)	0.935 (0.912-0.958)	0.966 (0.940-0.991)	0.937 (0.914-0.960)	0.972 (0.950-0.994)	0.938 (0.915-0.960)	0.971 (0.948-0.993)	0.942 (0.921-0.963)
NB	0.927 (0.887-0.966)	0.927 (0.905-0.950)	0.931 (0.893-0.969)	0.931 (0.909-0.952)	0.944 (0.910-0.977)	0.929 (0.907-0.951)	0.947 (0.915-0.980)	0.929 (0.906-0.951)

NN, Neural network; SVM, Support vector machine; RR, Ridge regression; LR, Lasso regression; NB, Naive bayes
