# Supplementary figures and images for: Metabolic signatures for gastric cancer diagnosis and mechanistic insights: a multicenter study
Source: EMBO Mol Med. 2025 Oct 27;17(12):3355–76. doi: 10.1038/s44321-025-00325-0 (PMC12686445; doi:10.1038/s44321-025-00325-0)

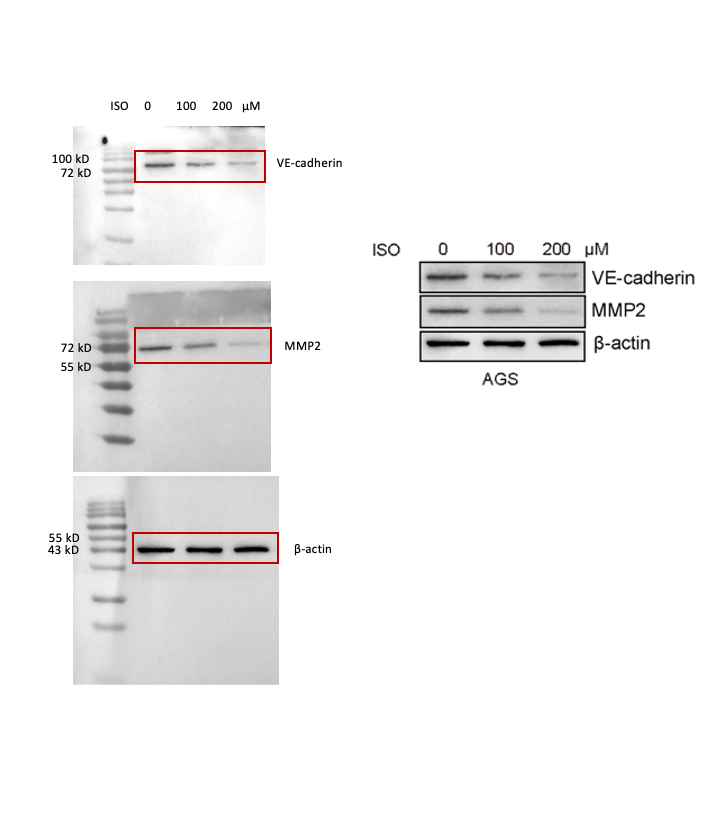

Supplement: Supplementary file 7 — Source data Fig. 6 [file 44321_2025_325_MOESM7_ESM.zip › EMM-2025-21992_Figure 6/EMM-2025-21992_Figure 6D/EMM-2025-21992_Figure 6D.tiff]

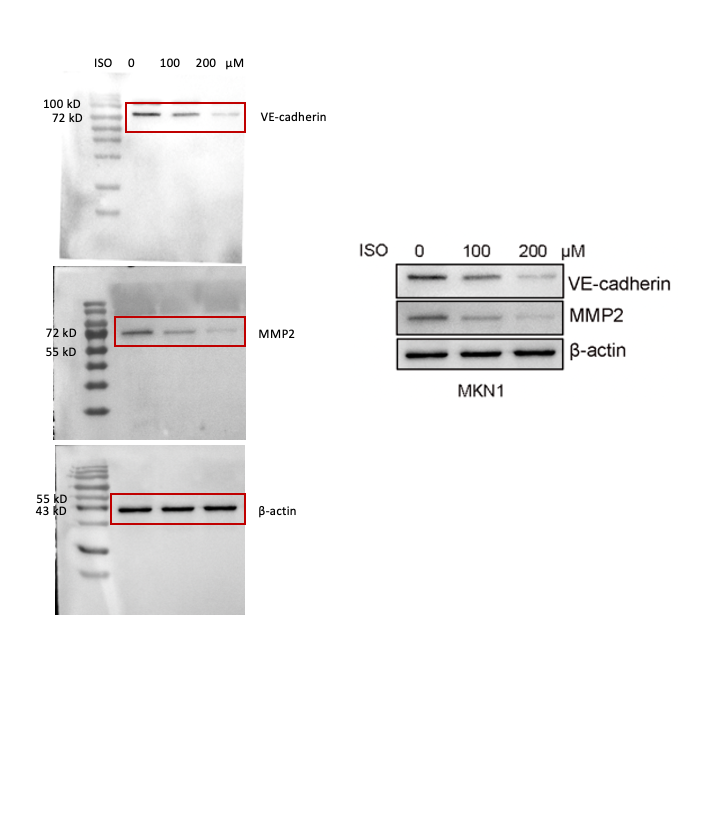

Supplement: Supplementary file 7 — Source data Fig. 6 [file 44321_2025_325_MOESM7_ESM.zip › EMM-2025-21992_Figure 6/EMM-2025-21992_Figure 6E/EMM-2025-21992_Figure 6E.tiff]

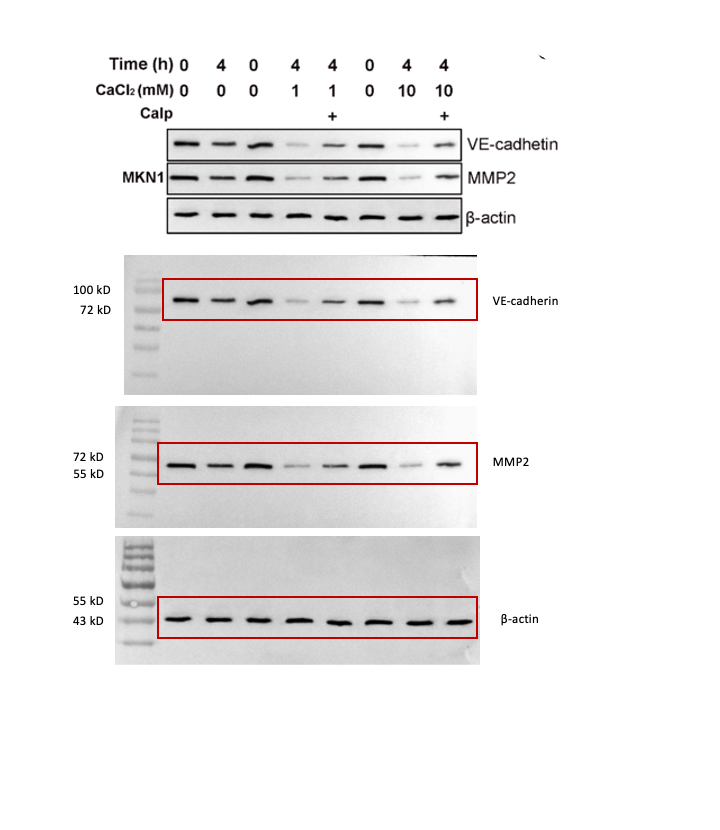

Supplement: Supplementary file 8 — Source data Fig. 7 [file 44321_2025_325_MOESM8_ESM.zip › EMM-2025-21992_Figure 7/EMM-2025-21992_Figure 7B/EMM-2025-21992_Figure 7B.tiff]

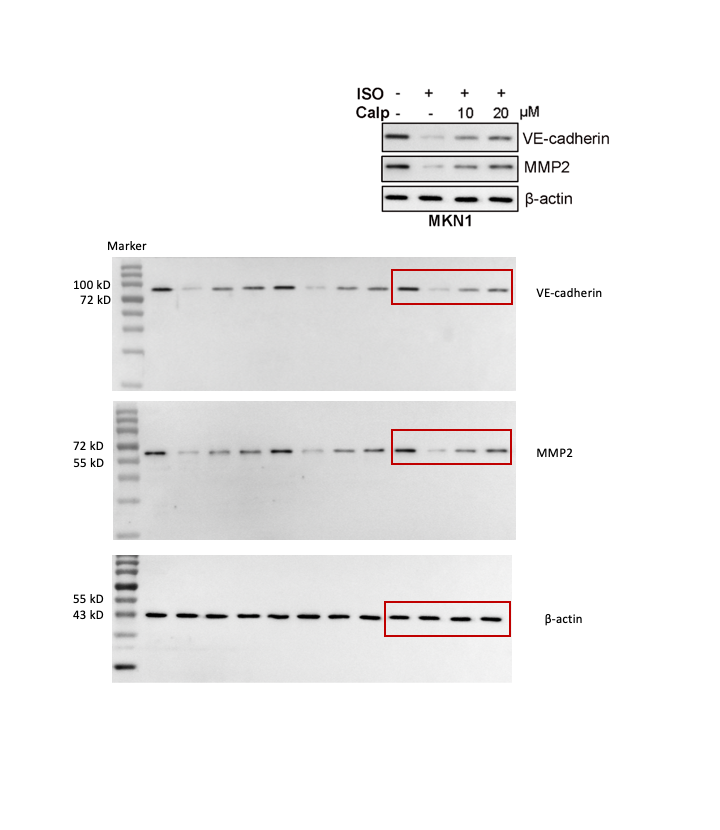

Supplement: Supplementary file 8 — Source data Fig. 7 [file 44321_2025_325_MOESM8_ESM.zip › EMM-2025-21992_Figure 7/EMM-2025-21992_Figure 7D/EMM-2025-21992_Figure 7D.tiff]

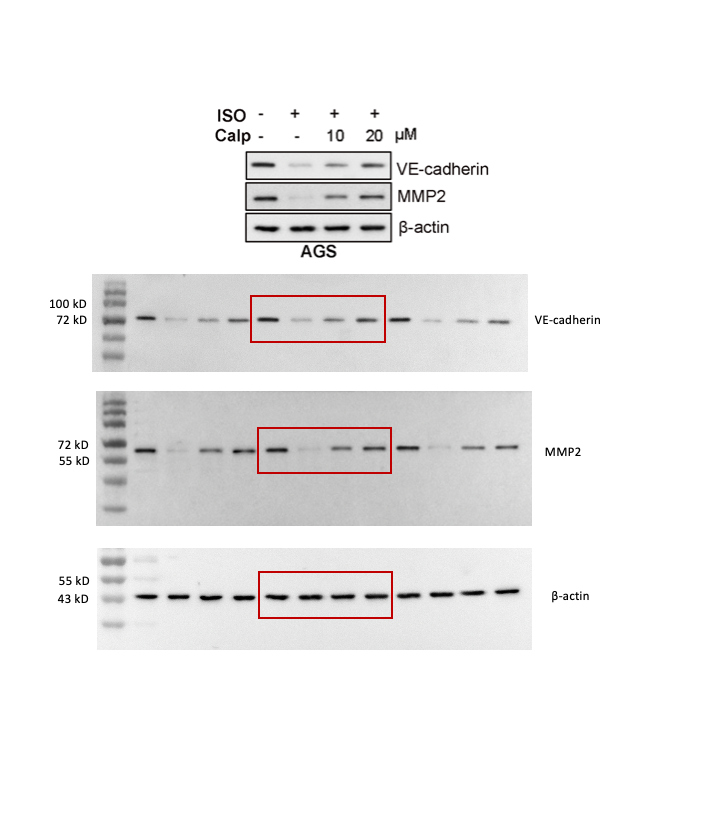

Supplement: Supplementary file 8 — Source data Fig. 7 [file 44321_2025_325_MOESM8_ESM.zip › EMM-2025-21992_Figure 7/EMM-2025-21992_Figure 7C/EMM-2025-21992_Figure 7C.tiff]
